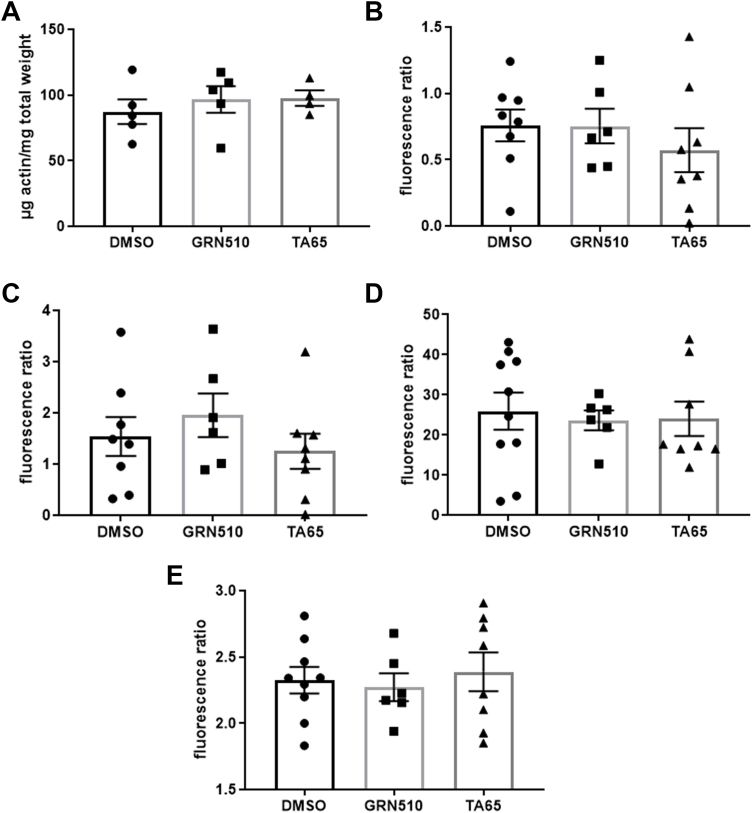# Increased telomerase improves motor function and alpha-synuclein pathology in a transgenic mouse model of Parkinson’s disease associated with enhanced autophagy

**DOI:** 10.1016/j.pneurobio.2020.101953

**Published:** 2021-04

**Authors:** Tengfei Wan, Emma J. Weir, Mary Johnson, Viktor I. Korolchuk, Gabriele C. Saretzki

**Affiliations:** a Biosciences Institute, Newcastle University, Campus for Ageing and Vitality, Newcastle upon Tyne NE4 5PL, UK; b Translational and Clinical Research Institute, Newcastle University, Campus for Ageing and Vitality, Newcastle upon Tyne NE4 5PL, UK

**Keywords:** Telomerase, Telomerase activator, Parkinson’s disease, Transgenic mouse model, Motor function, Alpha-synuclein, Autophagy

## Abstract

Protective effects of the telomerase protein TERT have been shown in neurons and brain. We previously demonstrated that TERT protein can accumulate in mitochondria of Alzheimer’s disease (AD) brains and protect from pathological tau in primary mouse neurons. This prompted us to employ telomerase activators in order to boost telomerase expression in a mouse model of Parkinson’s disease (PD) overexpressing human wild type α-synuclein. Our aim was to test whether increased *Tert* expression levels were able to ameliorate PD symptoms and to activate protein degradation.

We found increased *Tert* expression in brain for both activators which correlated with a substantial improvement of motor functions such as gait and motor coordination while telomere length in the analysed region was not changed. Interestingly, only one activator (TA-65) resulted in a decrease of reactive oxygen species from brain mitochondria. Importantly, we demonstrate that total, phosphorylated and aggregated α-synuclein were significantly decreased in the hippocampus and neocortex of activator-treated mice corresponding to enhanced markers of autophagy suggesting an improved degradation of toxic alpha-synuclein. We conclude that increased *Tert* expression caused by telomerase activators is associated with decreased α-synuclein protein levels either by activating autophagy or by preventing or delaying impairment of degradation mechanisms during disease progression. This encouraging preclinical data could be translated into novel therapeutic options for neurodegenerative disorders such as PD.

## Highlights

• Telomerase activators (TA) increase *Tert* expression in brains of a PD mouse model.
• Activator treatment improves PD motor symptoms: gait and balance.
• Activators reduce different forms of alpha-synuclein in brains of transgenic mice.
• Decreased autophagy markers LC3 and p62 suggest a better protein degradation.
• Our preclinical data suggest a use of TA to ameliorate PD-like symptoms.

## 1 Introduction

### 1.1 Telomerase and its non-canonical function in brain

The enzyme telomerase is best-known for its role in maintaining telomeres in dividing cells through its catalytic activity requiring the telomerase reverse transcriptase (TERT) protein as well as the RNA component TERC (or TR).

However, there is evidence that the protein part TERT has telomere- and TERC-independent non-canonical functions. One such non-canonical function is the localisation of TERT within mitochondria where it decreases reactive oxygen species (ROS) and protects cells from oxidative stress as well as apoptosis, in particular under conditions of increased oxidative stress (Ahmed et al., 2008; Haendeler et al., 2009; Sharma et al., 2012; Singhapol et al., 2013).

Some of these non-canonical functions of TERT can also protect neurons and the brain under normal physiological conditions (Miwa et al., 2016; Eitan et al., 2016). Pioneering studies from Mark Mattson’s group already suggested a role of telomerase in brain development and resistance against neurodegenerative agents such as amyloid beta in cultured mouse neurons (Zhu et al., 2000; Fu et al., 2002). Telomerase activity, characteristic for canonical function, is quickly downregulated at early developmental stages and not detected in adult human and mouse brain (Ishaq et al., 2016; Klapper et al., 2001). However, TERT protein persists in adult brain and exerts a non-canonical function. Telomerase activators in general can affect both, canonical and non-canonical functions of telomerase and TERT depending on the tissue/cell type analysed and whether telomerase activity is present or not. In brain, mainly the non-canonical role of TERT is analysed. Esther Priel’s group demonstrated a protective role of a synthetic telomerase activator in a mouse model of Amyotrophic lateral sclerosis (ALS) (Eitan et al., 2012) and in primary neurons against amyloid beta toxicity (Baruch-Eliyahu et al., 2019). The same group identified glutamate stress in neurons as a trigger for mitochondrial TERT localisation (Eitan et al., 2016). Iannilli et al. (2013) showed that a large fraction of cytoplasmic TERT forms a complex with RNA granules in adult neurons binding p15 mRNA which is released upon cellular stress.

Our group found more TERT protein in mitochondria of hippocampal CA1 neurons of Braak 6 stage Alzheimer’s disease (AD) brains compared to brains from age-matched healthy controls (Spilsbury et al., 2015). Although TERT protein and pathological tau excluded each other in neurons from AD brains, the functional consequences of mitochondrial TERT localisation are not clear yet. Using cultured primary mouse neurons with and without *Tert* expression we confirmed its protective function against pathological tau (Spilsbury et al., 2015). Neurons from *Tert* -/- mice (Chiang et al., 2004) had higher levels of mitochondrial superoxide in basal dendrites and higher lipid peroxidation in the soma compared to neurons from wild type littermates. Oxidative stress promoted mitochondrial localisation of Tert protein in neurons from wild type mice (Spilsbury et al., 2015).

In addition, we demonstrated in another study on dietary restriction and rapamycin, which both decrease the mammalian target of rapamycin (mTOR) signalling, that TERT protein localises to brain mitochondria and the presence of TERT was associated with a decrease in ROS both in a cell model as well as in mouse brain (Miwa et al., 2016). Moreover, a physical and functional interaction of TERT with mTOR signalling and autophagy including a possible existence of mTOR and TERT in a complex has been demonstrated previously (Kawauchi et al., 2005; Sundin et al., 2013; Ali et al., 2016; Miwa et al., 2016).

### 1.2 A transgenic mouse model of PD

These beneficial effects of TERT protein in the brain prompted us to use two telomerase activators in old mice as well as in a mouse model of Parkinson’s disease (PD) (Masliah et al., 2000). This transgenic mouse model (line D) was characterised extensively by others previously for neurodegeneration such as decrease in dopamine levels and tyrosine hydroxylase (TH) activity as well as compromised neuronal function due to the over-expression of human α-synuclein (Masliah et al., 2000; Amschl et al., 2013). This model shows a number of behavioural symptoms found in PD patients. In particular, motor functions such as changes in stature, gait and balance are known to be increasingly compromised during disease progression (Morris et al., 1994; Galna et al., 2015). In addition, there are also non-motor symptoms involved in PD such a mild cognitive impairment which can eventually result in PD related dementia (PDD) (Martin-Ruiz et al., 2019).

### 1.3 Telomerase activators

TA-65 is a highly purified extract from Astragalous membranaceous (Bernardes de Jesus et al., 2011) and GRN510, a small molecule based on cycloastragenol (Le Saux et al., 2013). Both telomerase activators have been suggested and analysed as anti-ageing treatments for extending very short telomeres in mouse and human studies (Bernardes de Jesus et al., 2011; Le Saux et al., 2013; Dow and Harley, 2016; Salvador et al., 2016). Previous studies showed that both activators increase telomerase activity by increasing *Tert* expression resulting in a physiological extension of very short telomeres (Bernardes de Jesus et al., 2011). In human studies using TA-65 (a nutraceutical with a GRAS (generally considered as safe) status) either improvement of telomere length at very short telomeres in PBMCs (Salvador et al., 2016) or improvement of an age-related disease such as macular degeneration were demonstrated, however, in the latter study without providing any data on telomere length (Dow and Harley, 2016)·

### 1.4 The role of α-synuclein for brain pathology in PD and mechanisms of degradation

Toxic proteins such as pathological tau and different forms of α-synuclein (α-syn) are degraded in the brain. However, this degradation is diminished during ageing and in neurodegenerative diseases like Parkinson’s disease (PD) (Daniele et al., 2018; Bukhatwa et al., 2010). Misfolded and aggregated α-syn forms Lewy bodies (LB) that are thought to play a central role in PD pathogenesis. Intracellular homeostasis of α-syn requires the proper degradation of the protein by three mechanisms: chaperone-mediated autophagy (CMA), macroautophagy and the ubiquitin-proteasome system (UPS) (Lopes da Fonseca et al., 2015).

The latter is thought to be the dominant process for the clearance of short-lived, damaged and misfolded monomeric α-syn (Ciechanover and Kwon, 2015). Structural and functional changes to proteasomal subunits have been described in PD in the substantia nigra of sporadic PD cases (McNaught and Olanow, 2003). However, for higher order oligomers and aggregates of α-syn the narrow proteasome opening might be inaccessible, and these species have a higher dependency on autophagy for degradation (Ciechanover and Kwon, 2015).

Chaperone-mediated autophagy (CMA) is involved in the degradation of monomeric wild-type α-syn in neuronal cells and defects in CMA could be responsible for neuronal death in PD (Li et al., 2011). Reduction of the CMA markers Hsc70 and Lamp2A were identified in the substantia nigra (SN) of PD brains (Alvarez-Erviti et al., 2010). It is also known that CMA decreases with age due to a reduced Lamp2A expression (Loeffler, 2019).

Aggregation-prone substrates such as oligomeric α-syn resistant to both the UPS and the CMA can be degraded by macroautophagy (Yu et al., 2009). It selectively degrades misfolded protein aggregates, long-lived proteins and damaged organelles. Cargoes are segregated into autophagosomes and transported along the microtubule tracks towards the soma, where they fuse with lysosomes to form autolysosomes and are eventually degraded by lysosomal hydrolases. The mammalian target of rapamycin (mTOR) kinase negatively modulates autophagy (Ravikumar et al., 2010). Decrease in autophagy and up-regulation of mTOR has been found in brains from DLB (Dementia with Lewy bodies) cases as well as in the line D PD mouse model that we used in our *in vivo* analysis (Crews et al., 2010).

The aim of this study was to examine whether telomerase activators are able to boost *Tert* expression levels in mammalian brain and to ameliorate PD-related symptoms such as motor deficits in a mouse model of PD. We found that increased *Tert* expression improved motor functions such as balance and gait. Moreover, we aimed to elude any underlying molecular mechanisms. Increased *Tert* expression after using telomerase activators (TA) resulted in a reduction of different α-synuclein forms. Since recent studies have demonstrated that hTERT overexpression can improve autophagy in cellular models (Ali et al., 2016) we hypothesised that boosting mTert levels could improve autophagic degradation of α-syn in brains *in vivo*. This finding could form the basis for the development of new therapeutic strategies in delaying or ameliorating the progression of neurodegenerative diseases such as PD and AD.

## 2 Material and methods

### 2.1 Mouse strains and treatments

Line D mice were generated by Masliah et al. (2000) and purchased from QPS (Austria). They overexpress human wt *SCNA* under the control of a PDGF- promoter. Mice were bred from two line D males and C57BL6 females. Genotypes were determined as described previously (Masliah et al., 2000). Both sexes were used for treatment.

Mice were kept in the animal facility at Newcastle University under a 12h-day/night cycle. Ethical approval was granted from the local ethics committee (project license 60/4542). Treatments started at 4 months old with 25 mg/kg body weight/day TA-65 (Bernardes de Jesus et al., 2011) and 10 mg/kg body weight/day GRN510 (Le Saux et al., 2013). Both activators were supplied as powder by TA Sciences Inc. (USA) on the basis of a material transfer agreement. For the treatment, powdered diet (Special Diets Services, Witham) at 6 g/mouse/day was mixed with telomerase activator (TA) suspension in 0.33 % DMSO (v/v) or 0.33 % DMSO solution as control (6 ml each). The amount of activators was adapted to body weight changes during the experiment. Treatment continued for 14 months until the age of 18 months.

25 line D (15 females, 10 males) were used as controls receiving DMSO. 21 mice (10 females, 11 males) were treated with GRN510 and 13 mice (7 females, 6 males) were treated with TA-65. From these 59 mice, 8 mice (4 controls, 4 treated) were lost due to weight loss, sudden death, PD-unrelated diseases (5) or PD-related phenotypes (3) such as trembling, tremor or seizures. Thus, 51 mice were available for analysis at 18 months including 2 more that showed trembling and tremors at that age. All 51 mice were used for behavioural tests while for mitochondrial function, RNA/protein and immunostaining brain hemispheres were used (n = 102) and split between the different downstream read-out measures. Only 1 treated mouse was found with a liver tumour at 18 months while one control mouse died very early with a liver tumour. Thus, until 18 month with 14 months of treatment there was no increased cancer incidence found.

### 2.2 Tissue processing

Mouse brains were dissected into hemispheres after cervical dislocation. One half was snap-frozen in liquid nitrogen and stored at −80 °C. Another half was either freshly used for the AmplexRED® assay (Invitrogen, UK) or fixed in 4% paraformaldehyde in PBS, processed and embedded in paraffin for immunofluorescence staining.

### 2.3 Treatment of neuronal cultures with telomerase activators

Primary neurons were isolated from day 15 C57BL/6 embryos as described in (Spilsbury et al., 2015). Treatments started from DIV (days *in vitro*) 10 for the exclusive analysis of non-canonical functions of mouse TERT (mTERT) when telomerase activity was undetectable (Spilsbury et al., 2015).

GRN510 (500 nM) and TA-65 (10μM) were dissolved in DMSO and neurons treated for 48 h. DMSO was used for controls.

### 2.4 qPCR analysis

RNA was isolated (RNAeasy, Qiagen), and cDNA obtained by reverse transcription (Invitrogen). *Tert* expression was analysed by q PCR.

SensiFAST™ SYBR® Hi-ROX Kit (Bioline) was used in a Step One Plus cycler (Applied Biosystems). Annealing temperatures and primer sequences are listed in Table 1. Melting tempertaure analysis was performed from 68 °C to 95 °C to determine specificity of the products. The expression of target genes was normalised to the expression of the housekeeping gene *Gapdh*, and the results were calculated and reported with the format of comparative threshold cycle (2^−ΔΔCT^).

**Table 1:** Primers used in PCR and qPCR.

Name	Sequence	Annealing temperature
m*Tert* forward	5′-GGATTGCCACTGGCTCCG	68 °C
m*Tert* reverse	5′-TGCCTGACCTCCTCTTGTGAC	68 °C
m*Gapdh* forward	5′-GAACGGGAAGCTCACTGGC	62 °C
m*Gapdh* reverse	5′-GACAACCTGGTCCTCAGTGT	62 °C

### 2.5 Immunofluorescence staining and analysis

Sections were deparaffinised and rehydrated with standard methods and antigen retrieval performed with citrate buffer in a microwave. Sections were blocked with 10 % serum and 0.1 % BSA. Primary antibodies (see Table 2) were incubated overnight at 4 °C and secondary antibodies for one hour at room temperature. Since antibodies against total and phosphorylated human α-synuclein were both raised in rabbit the former was incubated as described above. Then a monovalent Fab Fragments (goat-anti-rabbit) (Jackson ImmunoResearch Inc., USA) was incubated with the sections overnight in order to label the first primary antibody with Fab fragments which changed the initial species identity from rabbit to goat. After that, a secondary chicken-anti-goat antibody was incubated with the sections to label the Fab fragments with the usual method as described above. Finally, sections were mounted in Vectashield® anti-fade mounting solution with DAPI (Vector Laboratories), images taken in a fluorescence microscope Leica DMi8 (Leica Microsystems) and images then analysed with ImageJ software.

**Table 2:** Antibodies used for Immunofluorescence and IHC staining.

Name	Type	Host	Dilution	Supplier
Anti-α-synuclein filament	Primary	Mouse	IF 1:200	Leica NCL-L-ASYN
Anti-α-synuclein [MJFR1]	Primary	Rabbit	IF 1:150	Abcam, ab138501
Anti-α-synuclein (Ser129 phosphorylated)	Primary	Rabbit	IF 1:150	Abcam, ab51253
Anti-α-synuclein	Primary	Rabbit	IHC, 1:300	Abcam ab2080
Anti-βIII-tubulin	Primary	Rabbit	IF 1:500 WB 1:1000	Abcam, ab18207
Anti-βIII-tubulin	Primary	Mouse	IF 1:1000	Abcam, ab78078
Anti-p62/SQSTM1 (C-terminus)	Primary	Guinea pig	IF, 1:500	Progen, GP62-C-WBC
Anti-LC3B	Primary	rabbit	IF, 1:200	Cell Signaling technology, #3868
F(ab’)2-Goat anti-rabbit AlexaFluor® 633	Secondary		IF 1:1000	Invitrogen, A-21072
Goat anti-rabbit AlexaFluor® 594	Secondary		IF 1:1000	Invitrogen, R37117
Goat anti-mouse AlexaFluor® 594	Secondary		IF 1:1000	Invitrogen, R37121
Chicken anti-rabbit AlexaFluor® 594	Secondary		IF 1:500	Invitrogen, A-21442
Goat anti-rabbit AlexaFluor® 488	Secondary		IF 1:1000	Invitrogen, A-11008
F(ab’)2-Goat anti-mouse AlexaFluor® 488	Secondary		IF 1:1000	Invitrogen, A-11017
Chicken anti-goat AlexaFluor® 488	Secondary (tertiary with Fab Fragments)		IF 1:1000	Invitrogen, A-21467
Fab Fragments	Secondary		IF 1:30	Jackson ImmunoResearch Inc., 111-007-003
AlexaFluor® 488 goat anti guinea pig	Secondary			Invitrogen, A-11073

For microscopy, at least five images per brain region per animal were taken. Each fluorescence signal was captured by an individual channel. Microscope settings (such as exposure) were maintained consistently identical for all samples stained with the same antibody. Captured images were analysed with ImageJ software (NIH, USA). A threshold was determined to subtract most of the background and prevent oversaturation in each channel. Subsequently, the determined thresholds for all channels were applied to all images from the staining with the same antibodies, and then the images were converted to RGB colour. The regions of interest were carefully selected and the intensity for each colour of the selected region was measured. The raw intensity of each antibody channel was eventually normalised by the intensity of DAPI from the same region and the results were presented as the ratio of antibody intensity/DAPI intensity. Mean intensity ratios of the five images captured from the same animal were used as the individual data for each animal and then these individual data were grouped by treatment types for statistics. All analysis was performed blindly and only afterwards the groups for each sample were revealed.

### 2.6 ROS levels in isolated mitochondria

Mitochondria isolation and mitochondrial ROS analysis (AmplexRED® assay, Invitrogen, UK) were performed as described previously (Miwa et al., 2014, 2016).

Complex I is able to generate ROS when electrons flow in either forward or reverse direction. Using Complex I-linked substrates pyruvate and malate, electrons flow through Complex I in the forward direction. Using rotenone in that condition blocks complex I which gets maximally reduced and stimulates the maximum rate of superoxide production by complex I (Quinlan et al., 2013).

For determination of reverse electron flow succinate is used as complex II substrate which donates electrons to the ubiquinone pool. Rotenone blocks the reverse electron flow to complex I. The difference in superoxide production rates before and after rotenone addition to succinate-supported mitochondria indicates the amount of superoxide produced by complex I via the reverse electron flow.

### 2.7 Behavioural tests

#### 2.7.1 Rota-Rod test

The accelerating rota-rod test mainly measures movement coordination (Deacon, 2013). One day before the real test, mice were trained once on a Rat and Mouse Rotarod (IITC Life Sciences WPI, UK) where rotation speed accelerates automatically with time. Mice that could not stay on the rod any longer fell down onto sensors whereby data (running time, distance and final speed) were recorded automatically and transferred to a spreadsheet. Three trials per mouse were performed.

#### 2.7.2 Stride length test

The stride length test was used to measure parameters similar to human gait tests (Galna et al., 2015). Mice got their palms on the back limbs painted: right - red and left- blue. Mice were set on a narrow path with white paper and allowed to walk from one end to the other. The sheet with the footprints was labelled and later analysed blindly. At least three distances between two footsteps from each side were measured with a ruler. For each mouse, the means of the stride length and the standard deviation of the length were calculated blindly and only at the end grouped. A similar test setting was used in the bradykinesia test, just without painting of the feet. Mice were allowed to walk a 1-metre distance on a piece of clean paper and time was recorded.

### 2.8 Statistical analysis

All statistical tests were performed using SigmaPlot (Systat Software Inc.USA). One-way ANOVA was used for multiple-group comparisons and *t*-test was used for two-group comparisons. If the data was normally distributed, they were shown as bar graphs with mean ± SEM, and the significance presented as: * for P < 0.05, ** for P < 0.01, *** for P < 0.001, **** for P < 0.0001. Not normally distributed data were compared on ranks and shown as Box & Whisker plots with median and data from minimum to maximum. Post-hoc tests for both ANOVA methods were performed with Holm-Sidak test for normally distributed data, ANOVA on ranks was performed with Tukey’s post-hoc test for non-normally distributed but equal-group-sized data, and Dunn’s test for data that were neither normally distributed nor equal-group-sized.

## 3 Results

### 3.1 Telomerase activators increase *Tert* expression in mouse brain and isolated neurons

While *Tert* expression decreases in mouse brain during ageing (Klapper et al., 2001; Miwa et al., 2016) treatment of 24 months old female wild type (WT) mice with GRN510 and TA-65 for 3 months resulted in a significant increase in *Tert* expression for both activators (Suppl. Fig. 1A). We analysed balance and motor coordination using a static rod test for activator-treated 27 months old mice compared to young mice and old mice treated with DMSO only. We found a substantial decrease in the performance of old compared to young mice (Suppl. Fig. 1B). However, treatment with GRN510 was able to improve the performance of the old mice comparable to the level of young mice while for TA-65 there was a trend towards an improvement (Suppl. Fig. 1B).

To confirm that indeed neurons responded to the activator treatment with an increase in *Tert* expression, we treated cultured primary embryonic WT neurons with the two activators for 48 h. The chosen concentrations (500 nM GRN510 and 10 μM TA-65) increased *Tert* expression significantly (Suppl. Fig. 1C and D).

In order to exclude that treatment with the activators resulted in any changes of telomere length we hybridised brain sections with a telomere probe and analysed telomere length in pyramidal neurons from hippocampal CA1. As can be seen in Supplementary Fig. 2 there were no differences detected between the three groups.

In order to evaluate whether TA treatment is able to modify symptoms of PD, we employed a mouse model of Parkinson’s disease that overexpresses human WT *SNCA* under the human PDGF promoter (line D) (Masliah et al., 2000). These mice show behavioural deficits resembling PD symptoms such as compromised movement and coordination as well as α-syn-related brain pathology (Masliah et al., 2000; Amschl et al., 2013). Mice were treated from 4 months old with the same dosage of TA as published previously (Bernardes de Jesus et al., 2011; Le Saux et al., 2013) on a daily basis until 18 months.

**Fig. 1:**
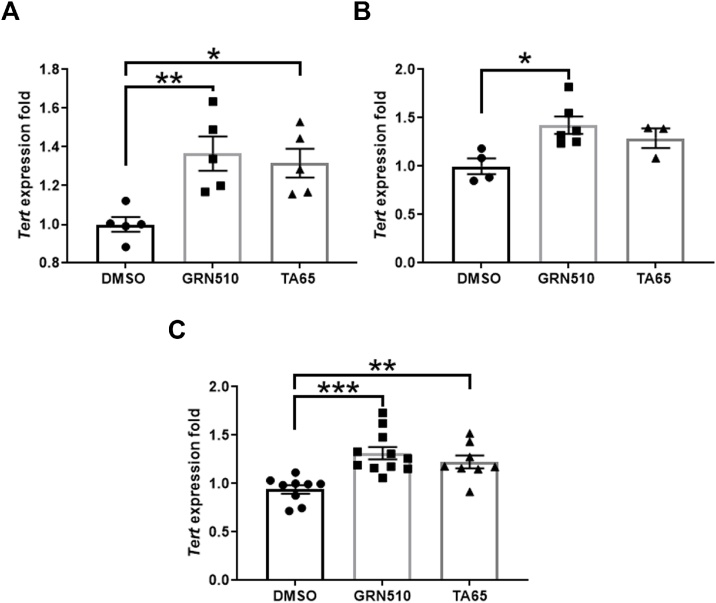
***Tert* expression in brains from line D mice after treatment with telomerase activators.** **A:** females (n = 5 per group), **B:** males, n = 4, 6, 3 for DMSO, GRN510 and TA65. **C:** pooled sexes. DMSO = 9, GRN510 = 11, TA-65 = 8. Results are presented as folds of the DMSO group based on 2^−ΔΔCT^ and bars represent means ± SE. Statistical analysis was performed by one way ANOVA with a Holm-Sidak as post-hoc test. * p < 0.05, ** p < 0.01, ***p < 0.001.

While whole brains from female mice showed an increased *Tert* expression for both activators (Fig. 1A), in males only GRN510 showed a statistically significant increase in *Tert* expression with a trend towards increase in TA-65 treated male mice (Fig. 1B). Combining both sexes resulted in a significantly increased *Tert* expression for both treatments (Fig. 1C).

### 3.2 Analysis of behavioural parameters relevant to PD

**Fig. 2:**
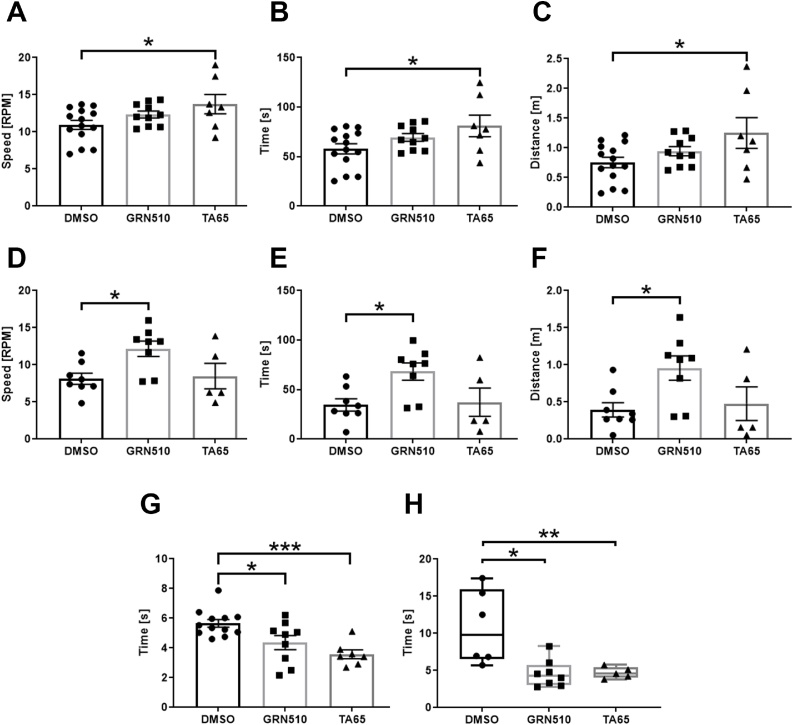
**Rotarod performance and walking speed analysis of TA treated line D mice.** Rotarod performance is shown as maximal speed (**A, D**), time on the rod (**B, E**) and distance travelled (**C, F**) in females (**A-C**) and males (**D-F**). Females: DMSO = 14, GRN510 = 10, TA-65 = 7. Males: DMSO = 7 GRN510 = 8, TA-65 = 5, Bars in A-G present mean ± SEM. Statistics was performed using a One-Way ANOVA with a Holm-Sidak as post-hoc test. **G, H**: time of females and males spent on travelling along a one meter long path, respectively. **H** shows median with minimum to maximum. Statistics was performed using a One-Way ANOVA on ranks. Sample size **G:** DMSO = 12, GRN510 = 10, TA65 = 7. **H:** DMSO = 13, GRN510 = 10, TA65 = 7. *

Since compromised motor function is an important feature of PD we used a rota-rod test with an incremental increase in the rod speed. We recorded time on the rod, its maximal speed and distance travelled by the mice and found that in females only TA-65 increased the rod parameters statistically significantly (Fig. 2A–C) while in males only GRN510 increased these parameters significantly (Fig. 2D–F).

Bradykinesia and slower walking speed are known to be compromising symptoms of PD (Moustafa et al., 2016). We determined walking speed by measuring the time a mouse requires to walk a 1 m distance and found a significantly increased walking speed decreasing walking time in females (Fig. 2G) and in males with both activators (Fig. 2H).

**Fig. 3:**
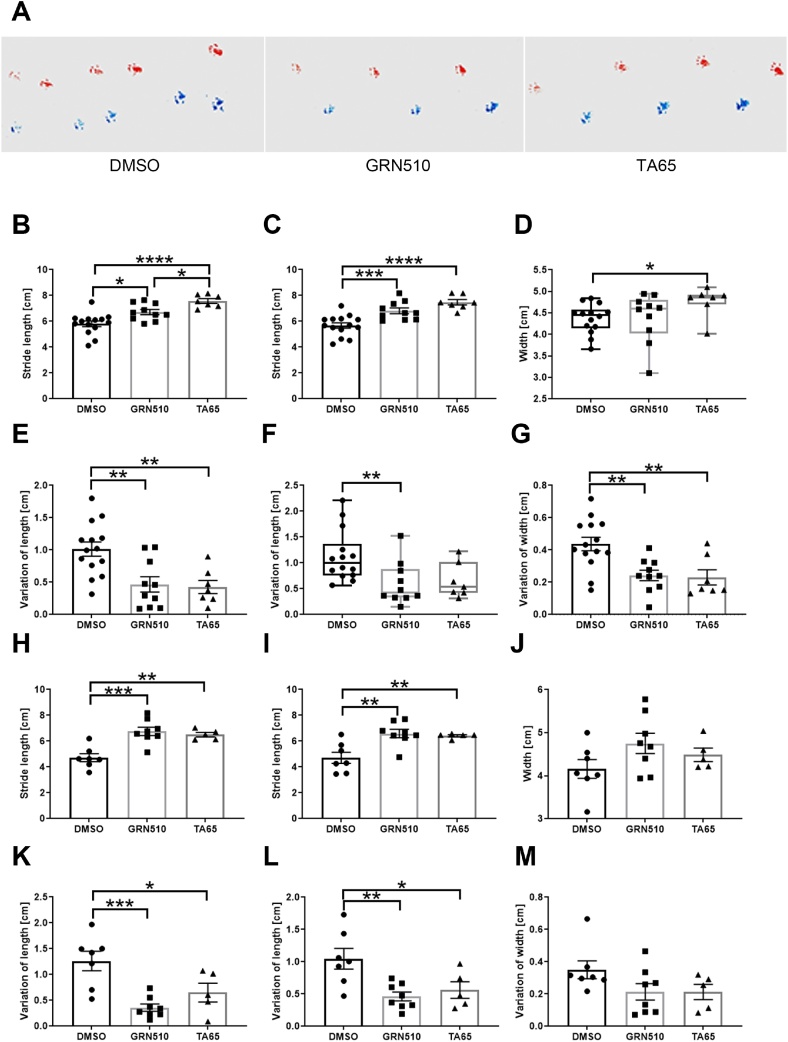
**Stride length and widths and their variation in telomerase activator treated line D mice.** **A:** Representative stride pattern of a DMSO, GRN510 and TA65 treated mouse. Graphs **B, C**, and **H, I** show the actual length in centimetres [cm] of strides from left and right feet of females (DMSO n = 14, GRN510 n = 10, TA65 n = 7) and males (DMSO n = 7, GRN510 n = 8, TA65 n = 5), respectively. Data are presented as mean ± SEM. **E, F** and **K, L** show the variation of stride length for the left and right legs for females and males, respectively. Standard deviation of the actual stride length data was used to calculate the variation of the stride length. Data for variation are shown as mean ± SEM of the stride length SD. **D** and **J** show gait width in females and males, respectively. **G** and **M** show the variation in width from females and males, respectively. Analysis was performed in the same way as for variation in stride length. Statistics was performed using a One-Way ANOVA with a Holm-Sidak as post-hoc test. * p < 0.05, ** p < 0.01, ***p < 0.001, **** p < 0.0001.

In addition to walking speed, irregular gait pattern is an important clinical characteristics of PD (Galna et al., 2015). We performed a simple stride length test measuring stride length and width of both hind legs as well as their variation (Fig. 3). Fig. 3A shows representative stride patterns for treated and untreated mice. For females, the stride length of both feet (left and right) as well their variations were highly significant for both telomerase activators (Fig. 3B, C, E andF). For stride width however, only TA-65 showed a significant increase (Fig. 3D) while both activators decreased variability (Fig. 3G). Similar results were obtained for males who also showed an increase in stride length (Fig. 3H and I) and a decrease in variability for both legs upon TA treatment (Fig. 3K and L). In contrast to the results in females, the width of strides and their variation in males were not significantly improved due to activator treatment (Fig. 3J and M).

In order to exclude the possibility that feeding line D mice with TA had any influence on body weight we weighed the mice weekly and there were no differences in body weight in either of the sexes under the treatment (Suppl. Fig. 3A and B).

We were also interested in examining other activity parameters such as rearing behaviour in a rearing test (Fleming et al., 2004; Gellhaar et al., 2015). Both activators increased rearing performance in females, while males were in general less active than females and there was no treatment effect (Suppl. Fig. 4A and B).

Since PD is frequently associated with mild cognitive impairment which can eventually lead to dementia, we performed a test to assess cognition in our mouse model after the treatment with TA. Since these mice have a compromised motor behaviour, locomotion-associated memory tests such as maze test were not suitable. Instead, we used a novel object recognition test (NOR) (Grayson et al., 2015). This test measures curiosity and memory in recognising a novel object compared to one which the mouse has seen before (“old”). Both activators improved performance in females (Suppl. Fig. 5A) while in males only GRN510 increased novel object recognition significantly with TA-65 having no effect at all (Suppl. Fig. 5B).

### 3.3 ROS release from isolated brain mitochondria

**Fig. 4:**
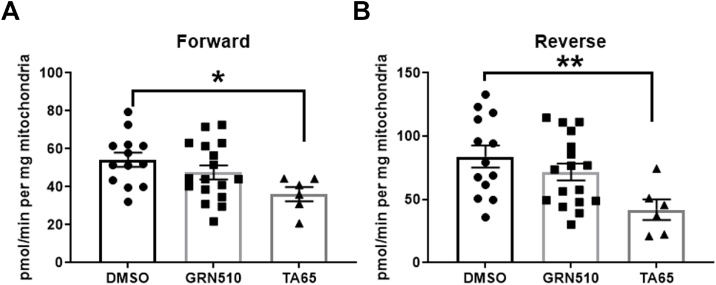
**ROS levels from and to complex I of brain mitochondria from treated line D mice.** **A:** Maximum capacity of Complex I ROS release from forward electron flow with Complex I substrates (pyruvate and malate) and rotenone, **B:** Capacity of Complex I ROS release from reverse electron flow from Complex II to complex I with Complex II substrate (succinate) and rotenone. Both sexes pooled, DMSO n = 13, GRN510 n = 17, TA65 n = 6. All data are normally distributed, analysed by One-way ANOVA and bars presented as means ± SEM. * p < 0.05, ** p < 0.01.

Due to our initial hypothesis and previous findings about a positive effect of telomerase within mitochondria decreasing levels of oxidative stress in brain tissue and cultured neurons (Miwa et al., 2016; Spilsbury et al., 2015) we measured hydrogen peroxide release in isolated brain mitochondria using an Amplex Red assay. In order to increase mouse numbers we pooled both sexes and used complex I substrate pyruvate/malate combined with rotenone treatment in order to determine maximal ROS release from complex I with forward electron flow (Fig. 4A). Maximal ROS release was significantly decreased with TA-65 as was reverse electron flow which uses the complex II substrate succinate together with rotenone as shown in Fig. 4B. In contrast, treatment with GRN510 did not show any effect in either forward or reverse electron flow.

### 3.4 Alpha-synuclein levels in hippocampus and neocortex

In order to evaluate whether increased *Tert* expression and improved balance and motor behaviour are reflected in changes of brain pathology we examined the levels of total, Ser^129^-phosphorylated as well as aggregated/filamentous α-syn in the CA1 and CA3 regions of the hippocampus and the neocortex. Since the transgenic model shows the highest α-syn levels in the olfactory bulb, hippocampus and neocortex (Amschl et al., 2013) we chose the latter two regions for analysis based on their significance for human PD pathology.

**Fig. 5:**
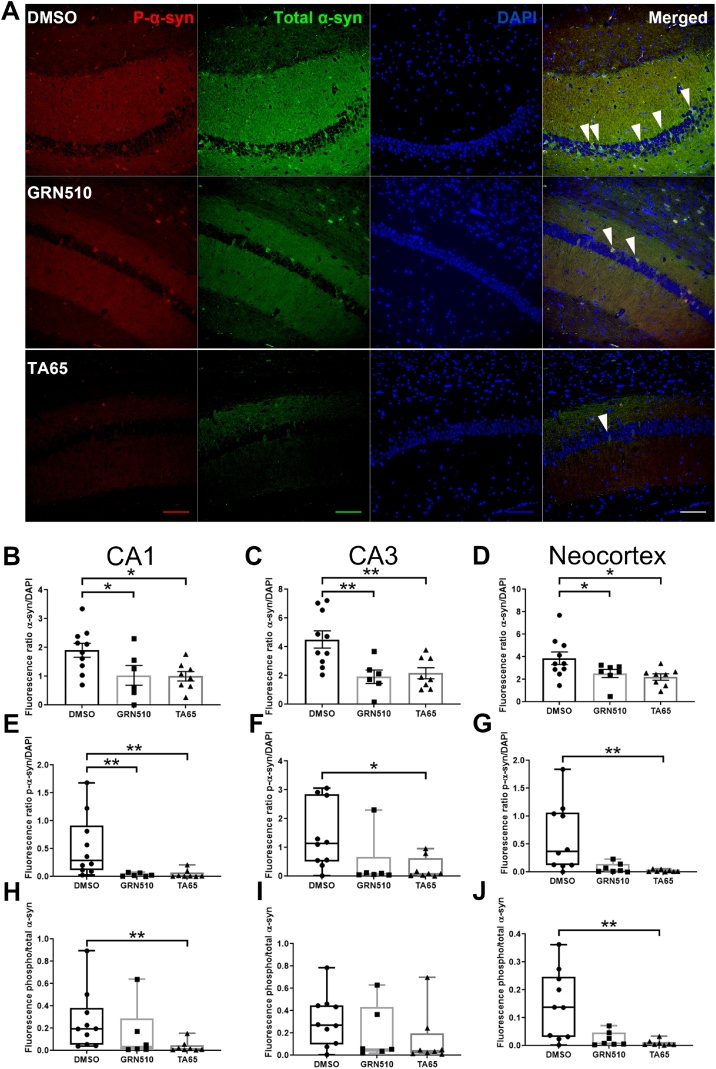
**Total and phosphorylated α-synuclein in different brain regions of TA treated line D mice.** **A:** Representative image showing total (green) and phosphorylated (red) human α-synuclein immunofluorescence staining on hippocampus CA1 region under DMSO, GRN510 and TA65. Scale bar represents 100 μm at 400x magnification. The white arrow heads in each merged image point out representative cells with both phosphorylated and total α-synuclein signal. **B,**C**,**D**:** Total α-synuclein levels related to DAPI nuclear signal in the whole area of the CA1 and CA3 pyramidal neuronal layer and neocortex. E**,F,**G**:** Phosphorylated α-syn levels related to DAPI nuclear signal in the whole area of the CA1 and CA3 pyramidal neuronal layer and neocortex H**,**I**, J:** Phosphorylated α-syn levels related to total α-synuclein in the whole area of the CA1 and CA3 pyramidal neuronal layer and neocortex. The data in B-Dare normally distributed, analysed with One way ANOVA and presented as mean ± SEM. * p < 0.05, ** p < 0.01. All remaining data are not normally distributed and shown in Box & Whiskers plot as median, minimum and maximum. Data has been analysed with a Kruskal-Wallis One Way Analysis of Variance on Ranks Multiple Comparison Procedure (Dunn's Method) Sample size: DMSO n = 10, GRN510 n = 6, and TA65 n = 8. * p < 0.05, ** p < 0.01.

Fig. 5A shows a representative image from immuno-fluorescence staining in the hippocampal CA1 region with antibodies against total α-syn and Ser^129^-phosphorylated α-syn. For CA1 we measured the fluorescence in the whole neuronal pyramidal layer. Treatment with both activators decreased total α-syn level normalised to DAPI signal significantly for CA1, CA3 and neocortex (Fig. 5 B, C and D). In contrast, phosphorylated α-syn normalised to DAPI signal was only significantly decreased by both activators in CA1 while in the hippocampal region CA3 and neocortex only TA-65 showed a significant decrease (Fig. 5E, F and G). Importantly, in the CA1 region of the hippocampus after TA treatment there was almost no phosphorylated α-syn detectable compared to DMSO treated controls while there was still around half the amount of total α-syn compared to controls. This result suggests that TA treatment might specifically prevent α-syn phosphorylation in that brain area. In order to characterise whether the decrease in phosphorylated α-syn is in general only due to the lower level of total protein, we also examined the ratio of phosphorylated to total α-syn level. While there was no specific decrease for phosphorylated α-syn in the CA3 region, both the CA1 region and the neocortex showed a specific decrease in phosphorylated α-syn related to total α-syn levels for TA-65 treatment, but not for GRN510 (Fig. 5H, I and J). In contrast, in other brain regions such as the CA3 of the hippocampus, the amount of phosphorylated α-syn seems to decrease rather proportionally to the total level of α-syn.

In order to complement immuno-fluorescence (IF) staining, we also used immuno-histochemistry (IHC) for total α-syn with a different α-syn antibody (see Table 2) as shown in Suppl. Fig. 6A. We evaluated here the layer of pyramidal neuronal bodies only by counting α-syn positive nuclei related to total nucleus number and found a significant decrease of α-syn positive pyramidal neuronal bodies for TA treated mice (Suppl. Fig. 6B). In order to have comparable results from the IF staining we examined the same region of pyramidal cells for the CA1 region. Supplementary Fig. 6C–E demonstrates a comparable significant decrease in total as well as phosphorylated α-syn normalised to DAPI in the same layer of neuronal bodies while phospho-α-syn normalised to total α-syn was significantly lower only for the treatment with TA-65. These results match well those for the whole neuronal area of CA1 described above (Fig. 5B, E and H) and demonstrate that the results of a decrease in total α-syn are independent of the staining technique and antibody used. For most results presented in Fig. 5 a larger data spread is found in the DMSO group than in the 2 treatment groups. This can be explained by a higher heterogeneity which is well known during the ageing process (Bahar et al., 2006).

**Fig. 6:**
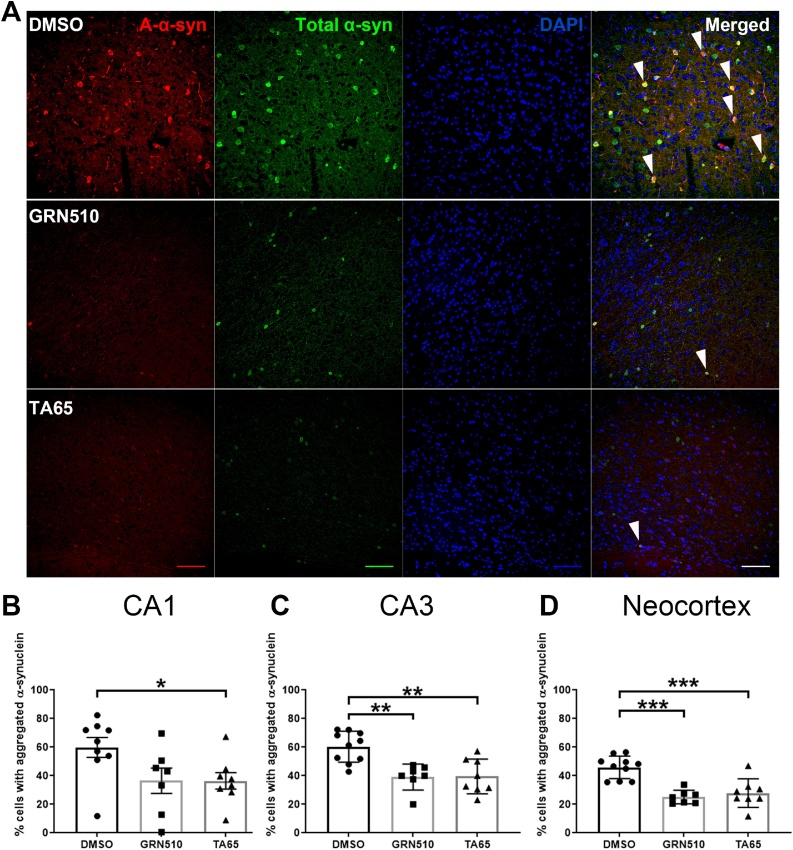
**Total and aggregated human α-synuclein in different brain regions of TA treated line D mice.** **A:** Representative image showing total (green) and filamentous (red) human α-syn immunofluorescence staining on the neocortex region under DMSO, GRN510, and TA65 treatment. The scale bar represents 100 μm at 400 magnification. White arrow heads in each merged image point out representative cells with both filament/aggregated and total α-synuclein signal. **B, C, D:** Percentage of cells with aggregated α-synuclein among total α-synuclein positive cells in CA1, CA3 and neocortex of line D mice at 18 months under TA treatment. Data from all regions are normally distributed, analysed by ANOVA and Holm-Sidak method as post-hoc test. Bars show mean ± SEM. Sample size: DMSO n = 10, GRN510 n = 6, and TA65 n = 8. * p < 0.05, ** p < 0.01, ***p < 0.001.

Analysing the percentage of cells with aggregated α-syn among cells with total α-syn (Fig. 6) we found that both activators decreased aggregates in the CA3 hippocampal region and the neocortex (Fig. 6C and D). However, in the hippocampal CA1 region only TA-65 treatment led to a significant reduction of aggregated α-syn while GRN510 treatment resulted in a similar trend without reaching statistical significance (Fig. 6B). The results demonstrate that aggregated α-syn is specifically decreased compared to the already lower total α-syn levels.

### 3.5 Is autophagy involved in the degradation of α-synuclein?

Since the human wild-type *SNCA* gene is constitutively expressed in the line D model, we focused on the analysis on the decrease of protein levels of the different α-syn forms. It was recently demonstrated that telomerase can increase and improve protein degradation mechanisms such as proteasomal activity and mTOR signalling/macroautophagy (Im et al., 2017; Ali et al., 2016). This prompted us to hypothesise that the increase in *T*ert levels might promote protein degradation mechanisms which degrade different forms of α-syn. While the proteasome degrades preferentially soluble, short-lived proteins, the autophagy process degrades primarily long-lived proteins or protein aggregates (Ciechanover and Kwon, 2015).

**Fig. 7:**
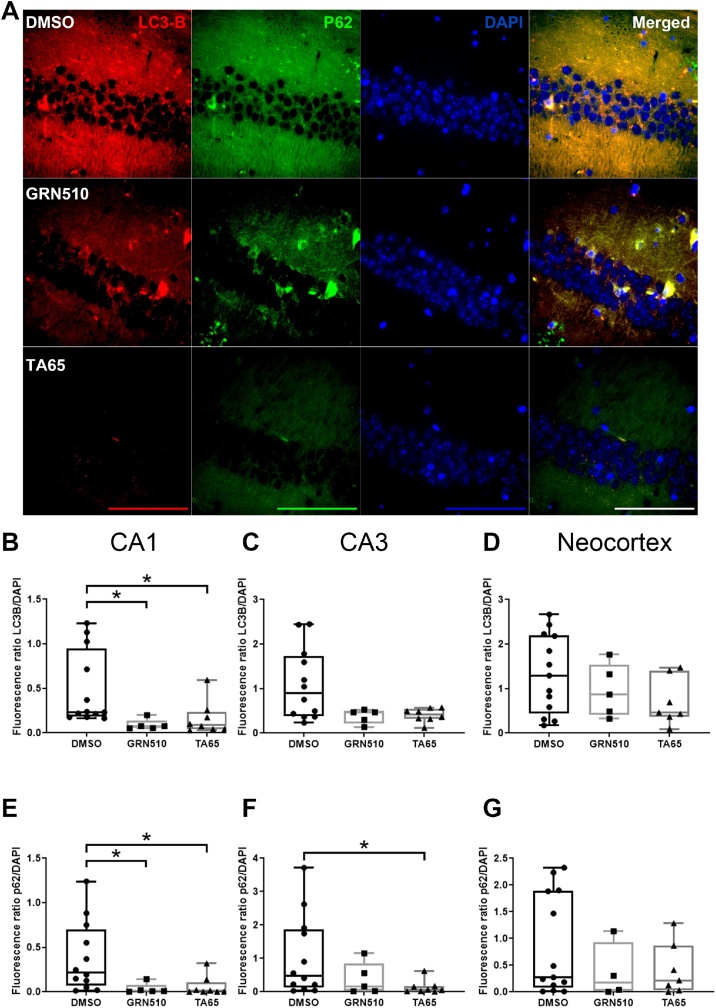
**Decreased LC3 and p62 proteins in different brain regions.** **A:** Representative fluorescence images (630x) of LC3 (red) and p62 (green) staining in the hippocampal CA1 region, nuclei stained by DAPI in blue. Scale bar shows 100 μm. **B-D:** LC3 fluorescence intensity related to DAPI signal in the 3 indicated brain regions. Sample size: N = 12, 5, 8 for DMSO, GRN510 and TA65 treated mice, respectively. * p < 0.05. **E-G:**p62 fluorescence intensity related to DAPI signal in the 3 indicated brain regions. Same sample size as in B-D, p<0.05.

In order to examine whether autophagy degradation can be involved in our experimental system after the treatment with telomerase activators, we analysed the levels of the established autophagy markers LC-3 and p62. Fig. 7A shows representative images for both proteins in the hippocampal CA1 region of DMSO, GRN510 and TA-65 treated mice.

During autophagy LC3-I is converted to LC3-II through lipidation by a ubiquitin-like system that allows for LC3 to become associated with autophagic vesicles and the LC3B antibody has a stronger reactivity with the lipidated, type II form of LC3.

As expected for the lipidated form of LC3, its staining showed a vesicular pattern in the control brains (Fig. 7A, upper row, left panel). We found a parallel significant decrease for both proteins, LC3B and p62, in the CA1 region for both activator types (Fig. 7B and E). However, for CA3 there was a significant difference for p62 only in TA-65 treated brains (Fig. 7F) while LC3B showed a tendency towards decrease upon activator treatment (Fig. 7C). In contrast, there were no significant differences in the fluorescence signals for both proteins in the neocortical area (Fig. 7D and G).

We can also exclude that general protein level in treated brains decreased since neither protein content of brain lysates (Suppl. Fig. 7A) nor other proteins (MnSOD, MTCO1, tyrosine hydroxylase and beta III tubulin) showed any changes between groups (Suppl. Fig. 7B–E). Although only CA1 showed significant results for both activators for LC-3B and p62 levels, the other two brain regions followed a very similar trend suggesting that the three regions might be rather similar for autophagy induction.

Together, our data suggest that increased *Tert* gene expression might promote the activity of autophagy as a well-known protein degradation process.

## 4 Discussion

Since for neurodegenerative diseases like PD and AD currently no cure is available the development of new therapies that ameliorate or delay symptoms is of utmost importance.

Transgenic mice over-expressing human *SNCA* have been established as useful tools in PD research and can also be employed for testing of novel treatment strategies.

Based on pilot experiments in old wild-type mice where treatment with telomerase activators increased *Tert* expression and improved balance, we used these activators in an established mouse model of PD (line D). These mice express the transgene mainly in the neocortex, hippocampus as well as the olfactory bulb (Masliah et al., 2000; Amschl et al., 2013) while the substantia nigra, like in other transgenic PD models, seems much less involved than in the human disease pathology. In accordance with the age-dependency of α-syn accumulation, transgenic α-syn in this model has been reported as increasing gradually during ageing and reaching its highest level around 12 months (Amschl et al., 2013). In parallel, these authors demonstrated a decrease in dopamine levels and a balance phenotype. Thus, even without the substantia nigra being a prominent target of transgenic α-syn pathology, the model seems to represent human PD features rather well.

Since neurons do not possess any telomerase activity at adult stages (Klapper et al., 2001; Ishaq et al., 2016) we did not expect any changes in telomere length. In order to exclude this effect we analysed telomere length in neurons from hippocampal CA1 and did not find any significant differences between the three groups. Thus, we are confident that the beneficial effects of the telomerase activators are based on non-canonical properties of the telomerase component TERT via a physiologically meaningful increase in the expression of the *Tert* gene.

Behavioural tests such as rota-rod, gait test and a rearing test as well as a memory task (novel object recognition) showed beneficial effects of 14 months treatment with telomerase activators. Corresponding to the previously described gradual increase in α-syn protein in the hippocampus and the striatum (Amschl et al., 2013) we detected typical PD symptoms such as tremor or trembling only starting at 12.5 months while most mice (90 %) did not show very obvious motor symptoms. The fact that all five mice (3 that died early and 2 that showed symptoms but were still included at 18 month) with severe PD symptoms were males is in good accordance with the known risk factor for male gender in humans (Taylor et al., 2007; Cereda et al., 2016). Simunovic et al. (2010) found that many genes were differentially regulated in DA neurons of brains from sporadic PD cases between genders including genes involved in mitochondrial oxidative phosphorylation and energy consumption, apoptosis and synaptic transmission which were more strongly downregulated in male patients and could all contribute to the underlying molecular mechanisms for a higher PD risk in males.

No increase in tumour incidence was found until the age of 18 months with a 14 months treatment period (data not shown). As expected, *Tert* expression was significantly increased in cultured neurons as well as due to activator treatment in line D mice with the exception that TA-65 in males did not reach statistical significance which could be due to a low mouse number in this group (n = 3).

A reflection of some gender specificity is also the finding that in the rotarod test TA-65 was only effective in females while in males only treatment with GRN510 improved balance and coordination significantly. In contrast, in the stride length/gait test almost all analysed parameters, including stride length for both hind legs as well as variation between strides for length showed significant improvement in both sexes. The only exception was stride width which did not show any significant improvements in males and for females only TA65 was significantly different from controls while variation in width was significant for both activators in females.

Gait disorders are featured symptoms in PD (Morris et al., 1994, 1998) and reduction in stride length and higher variation of stride length are the main characteristics of Parkinsonian gait (Blin et al., 1990; Hausdorff et al., 1998; Morris et al., 1998). Therefore, longer average length of the strides indicates a less severe PD gait symptom.

Since bradykinesia is also a commonly featured clinical symptoms for Parkinson’s disease (Clarke et al., 2016), we analysed walking speed of mice for 1 metre. While males walked in general slower than females, both telomerase activators increased walking speed significantly in both sexes.

Interestingly, we did not find any significant differences after TA treatment in general motor activity, measured as the number of rearings with front paws in a cylinder test, for male mice, while in females both treatments increased general physical activity. This is in line with females being more active than males and showing on average twice as many rearings on their hind legs.

Mild cognitive impairment is an important non-motor feature of PD in many patients. It eventually can even result in Parkinson’s disease related dementia (PDD). Since motor features (gait and walking speed) were compromised in our mouse model, we used a cognitive test that does not require extensive movement - the novel object recognition test. The interest of a mouse in a novel compared to an old object is a read-out for memory function. While male mice showed an increased interest in the novel object only after GRN510 treatment, in females both treatments successfully increased the interest for the new object compared to DMSO controls. The higher efficiency of GRN510 in males corresponds to that found in the rota-rod test as well as the higher *Tert* expression after GRN510 compared to TA65 in male mice.

In order to assess whether the treatment with telomerase activators improved mitochondrial function and decreased ROS levels from complex I, we analysed isolated brain mitochondria in an Amplex Red assay. Mitochondrial ROS such as superoxide generated during oxidative phosphorylation at respiratory chain complexes I-III are the main source of free radicals in most cells. In particular, complex I deficits are thought to have a central role in the pathogenesis of sporadic PD associated with WT α-syn (Dawson and Dawson, 2003). Impaired complex I activity and its correlated higher ROS levels were found in the substantia nigra regions and platelets of patients with sporadic PD (Schapira et al., 1994, Schapira et al., 1998). ROS produced via reverse electron transport (RET) at Complex I have been implicated in major signalling pathway between mitochondria and the rest of the cell (for review see Scialò et al., 2017). We have shown previously that dietary restriction decreased forward and reverse ROS production in brain mitochondria in a TERT-dependent manner (Miwa et al., 2016). This decrease in ROS was associated with increased TERT protein within brain mitochondria. Blocking TERT’s subcellular shuttling or deleting *T*ert blunted the effects of lowering ROS *in vitro* as well as *in vivo* (Miwa et al., 2016). Thus, we hypothesised that a similar process of TERT protein localisation due to increased *T*ert expression levels might be the underlying mechanism for the decrease in ROS. However, due to the lack of specific anti-TERT antibodies (Xi et al., 2015) that work in mouse tissue, we were not able to test this hypothesis directly.

In our study, the maximum ROS release, generated with forward and reverse electron flow from and to complex I was only significantly decreased in mitochondria from mice that were treated with TA-65 while GRN510 did not show any effect on ROS release. Consequently, we conclude that a ROS decrease from mitochondria is not the main mechanism causing the reduction of α-synuclein levels in our study. The preferential improvement of ROS levels after TA-65 treatment suggests differences between both activators with the natural compound TA-65 having possibly additional anti-oxidant properties compared to the synthetic GRN510.

TA-65 as a highly purified extract contains at least 5% non-telomerase activator compounds (Harley et al., 2011) which could also have antioxidant capacities. Another possibility is that more TERT is associated with higher MnSOD levels (Martens et al., 2019) or other antioxidants such as glutathione and peroxiredoxin as described previously for cultured cells (Indran et al., 2011). While we found a slight increase in the expression of *MnSOD* in TA-65 treated brains, this could not be confirmed on the protein level (data not shown). Thus, other antioxidants or methods of action might be involved and should be evaluated in future studies. An ideal method for excluding off-target effects of TA-65 would be to treat a transgenic mouse model of α-synuclein in a *Tert* knock-out background which was beyond the scope of our study.

In addition to behaviour and mitochondrial ROS levels we also assessed brain pathology. Here we mainly focussed on the determination of different forms of α-syn and their levels in previously described brain areas such as hippocampus and neocortex (Amschl et al., 2013) of treated mice. Strikingly, we found both in the neocortex and the CA1 and CA3 hippocampal regions that the treatment with telomerase activators significantly reduced the levels of total, phosphorylated and aggregated α-syn. In the hippocampal CA1 region we used both, immuno-fluorescence (IF) as well as immuno-histochemistry (IHC) and determined the number (IHC) and fluorescence amount for the layer of pyramidal neuronal bodies as well as the whole CA1 region using IF. In addition, for IF we were able to determine the fluorescence levels for different α-syn forms in the whole CA1 region. Both, total as well as Ser^129^ phosphorylated α-syn levels were significantly reduced when related to cell number (DAPI signal intensity). However, the ratio between the phosphorylated and total α-syn showed mainly a trend for a reduction and reached statistical significance only in the CA1 region for the treatment with TA-65. This additional reduction would suggest an additional decrease of protein modification which was not just related to reduced total α-syn protein. Similarly, aggregated α-syn was related to the amount of cells with total α-syn and showed a significant decrease in both neocortex and CA3 with both telomerase activators while in CA1 only TA-65 decreased the amount of aggregated α-syn significantly. This result suggests that protein degradation processes have most likely been activated in these regions.

The telomerase protein TERT has recently been shown to upregulate both UPS and autophagy in cellular models. Im et al. (2017) determined that TERT binds to multiple 20S proteasome subunits, stimulates proteasomal activity and promotes assembly of the 20S with the 19S subunits. Thus, the study identified a novel chaperone activity of TERT for the 26S proteasome assembly. Similarly, Ali et al. (2016) reported a direct stimulation of autophagy by TERT via mTOR suppression. The authors demonstrated that hTERT inhibits the kinase activity of the mTOR complex thereby activating autophagy. In contrast, *TERT* deficient cells were not able to properly execute an autophagic flux (Ali et al., 2016). These results add a novel non-canonical function for the TERT protein. Additionally, a physical complex formation between TERT and mTOR, an important regulator of autophagy, has been reported previously (Kawauchi et al., 2005; Sundin et al., 2013). While the ubiquitin-proteasome system (UPS) is mainly responsible for the degradation of monomeric proteins such as α-syn, autophagy engages in the degradation of aggregated neurodegenerative proteins such as α-syn and tau (Lopes da Fonseca et al., 2015; Mputhia et al., 2019). Our results of significantly decreased total, phosphorylated and aggregated α-syn suggest that some of these degradation processes might be involved. In order to test whether known players of autophagic degradation are involved in our system, we determined the levels of the autophagic protein LC3 and an autophagy substrate p62. We found a significant decrease of both proteins after the activator treatment in the CA1 region, while CA3 and neocortex displayed only a trend towards a decrease due to increased *Tert* levels. Although we do not know the reasons for those differences, all three analysed brain regions seem to show a comparable tendency. Interestingly, Gao et al. (2015) published that CA1 showed a higher autophagy upregulation together with an increased neuronal survival compared to CA3. Thus, it is possible that there exist physiological differences between those brain regions.

Accumulation of misfolded α-syn in dopaminergic neurons is an established contributor to PD which, in turn, can be facilitated by the impairment of the autophagy-lysosome pathway (Karabiyik et al., 2017; Klein and Mazzulli, 2018). The decrease in the autophagosomal markers LC3-II and p62 in α-syn-expressing tissues in response to telomerase activator treatment suggests an enhanced clearance of autophagic vesicles, which is in agreement with the clearance of α-synuclein.

p62 is the selective cargo receptor for autophagy facilitating degradation of misfolded proteins. It is involved in the formation of the autophagosome, the delivery of ubiquitinated proteins to the proteasome, and the formation of ubiquitinated aggregates for autophagic clearance. When autophagy is stimulated, p62 levels decrease. Consequently, the finding of a decrease in p62 levels in our system suggests an improved degradation of α-syn although we cannot specify that any further currently. Future studies should characterise the exact molecular mechanisms after TERT increase by telomerase activator treatment. However, based on our findings we hypothesise that the telomerase protein TERT might improve protein quality control and thereby protect neurons from toxic proteins such as α-syn in neurodegenerative diseases. A stimulation of autophagy with different agents resulting in decreased levels of aggregated a-syn has been described previously (Crews et al., 2010; Guo et al., 2016; Hu et al., 2017; Gao et al., 2019). Gao and colleagues (2019) demonstrated a correlation between α-syn and p62 levels. Crews et al. (2010) showed for the line D transgenic PD model that mTOR and LC-3 were increased compared to controls and both markers colocalised with transgenic α-syn. In agreement with our results, the study demonstrated that rapamycin treatment could decrease fibrillar/aggregated alpha-synuclein levels by activating autophagy. However, the role of TERT was not considered in that study. In addition, it was demonstrated previously that α-syn accumulation could have a negative impact on autophagy activity (Winslow et al., 2010). Therefore, increased clearance of α-syn in response to TERT activators could further upregulate autophagy and facilitate more clearance in a positive feedback loop. Moreover, another possibility is that activator treatment is able to prevent age-related impairment of autophagy leading to the same effect of less α-synuclein level. Since this is the first study on a connection between TERT, autophagy and α-syn, the underlying mechanisms linking these processes (including the characterisation of further autophagy markers) will have to be analysed in more depth in the future.

**Fig. 8:**
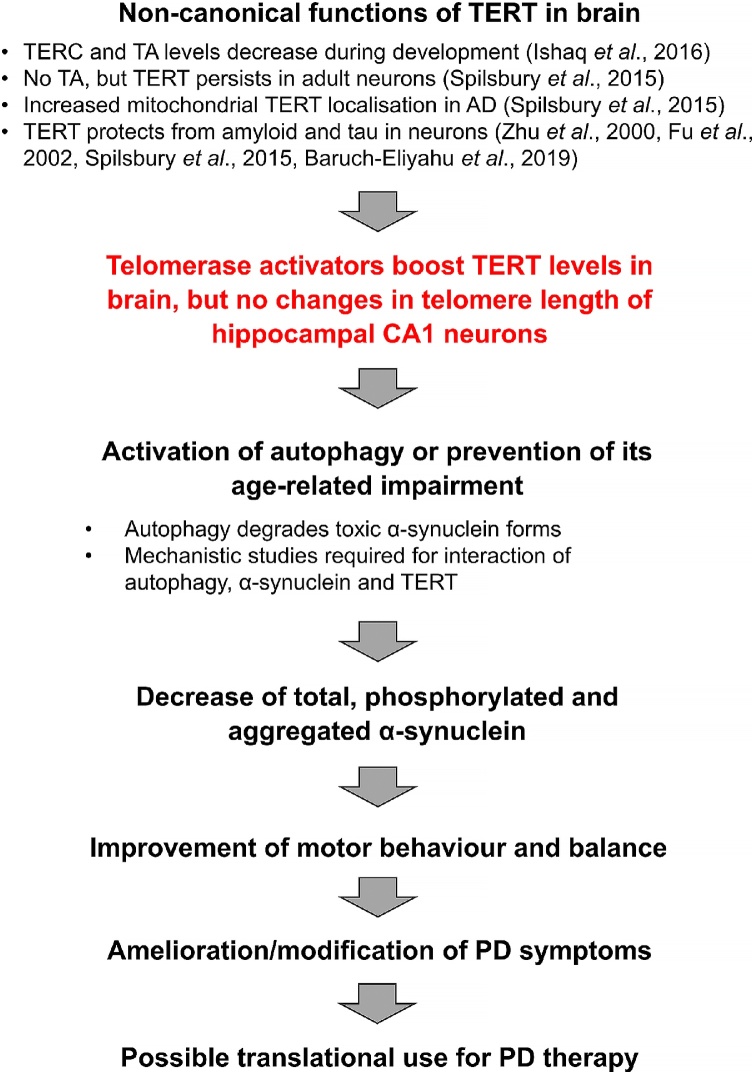
Scheme summarising findings of this study. TA: telomerase activity. AD: Alzheimer's disease.

In summary, our study suggests that treatment with telomerase activators, in particular the plant-based extract TA-65 which is commercially available and considered a safe nutraceutical, could be a valid strategy for ameliorating and/or delaying symptoms of PD. The scheme in Fig. 8 summarises our findings and an outlook into future research and application.

Studies using TA-65 have already been performed and beneficial effects observed in age-related macular degeneration and subjects with metabolic syndrome in humans (Dow and Harley, 2016; Fernandez et al., 2018) as well as extending short telomeres in mice and humans (Bernardes de Jesus et al., 2011, Salvador et al., 2016). Thus, in addition to extending the shortest telomeres via its canonical telomerase function, the increase of TERT in the brain could be a valuable anti-ageing intervention as well as a novel therapeutic strategy for modifying and ameliorating neurodegenerative diseases such as PD or AD.

Strikingly, both activators have slightly different effects with TA-65 being the more effective in various behavioural tests in our study. While both activators improved walking speed and gait parameters in both sexes, they had sex-specific effects in the rota-rod test where females improved their balance and motor coordination skills preferentially to TA-65 while GRN510 showed an effect only in males. While both activators reduced total α-synuclein levels, TA-65 resulted in more frequent reduction of phosphorylated and aggregated α-synuclein levels. From the three analysed brain regions, CA1 more frequently showed a positive effect in the reduction of various α-synuclein forms which correlated with a significant decrease in LC-3B and p62 levels.

Consequently, further mechanistic studies are required in order to establish i) that improvements in α-synuclein degradation are direct and specific consequences of higher TERT levels, ii) what causes the differential effects of both activators and iii) whether the activation of autophagy via increased *Tert* expression levels is a direct “on-target” effect of telomerase activators or mediated by additional molecular pathways.

## Funding

This study was funded by a Medical Research Council, UK (MRC Grant), (MC_PC_13071) to GS. The funding body was not involved in the design of the study, collection, analysis, interpretation of data or in writing the manuscript.

## Authors' contributions

TW performed most of the experiments and stainings, analysed data, composed the graphs and contributed to writing the manuscript, EW and MJ also performed stainings, VK contributed to the autophagy part and manuscript writing and GS designed the study, obtained funding and wrote the manuscript. All authors read and approved the final manuscript.

## Declaration of Competing Interest

The authors declare no competing interests.

## Figures and Tables

**Figure:**
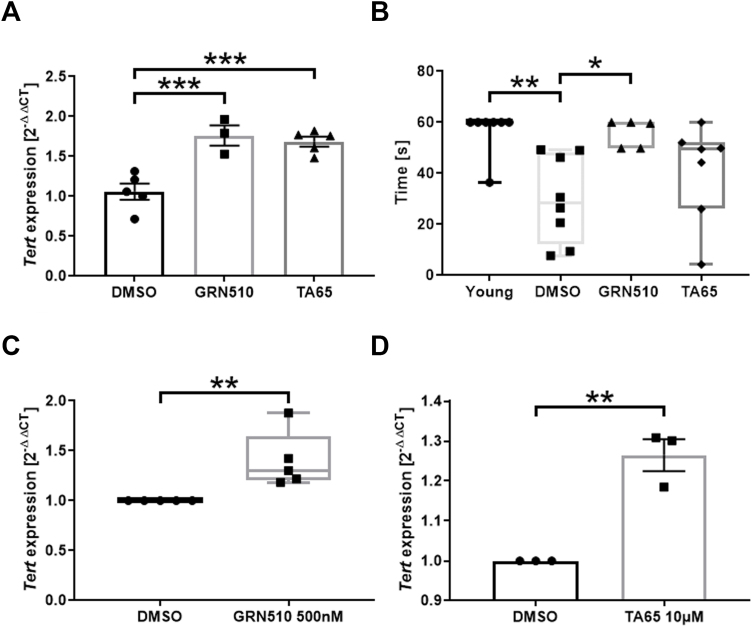


**Figure:**
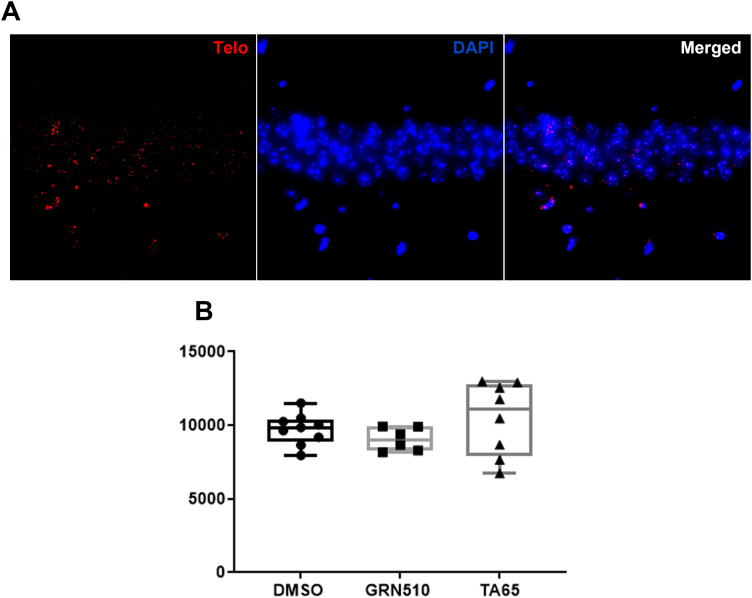


**Figure:**
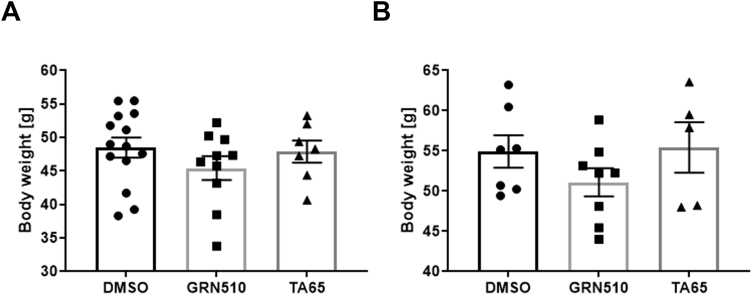


**Figure:**
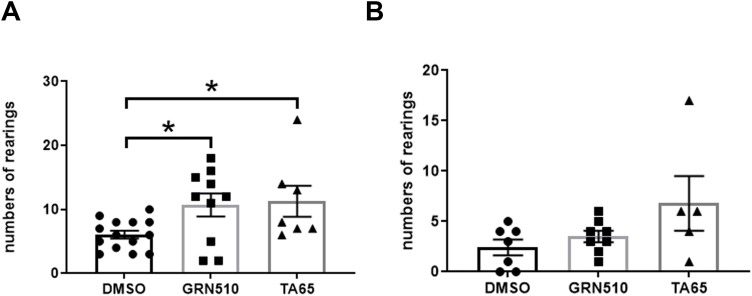


**Figure:**
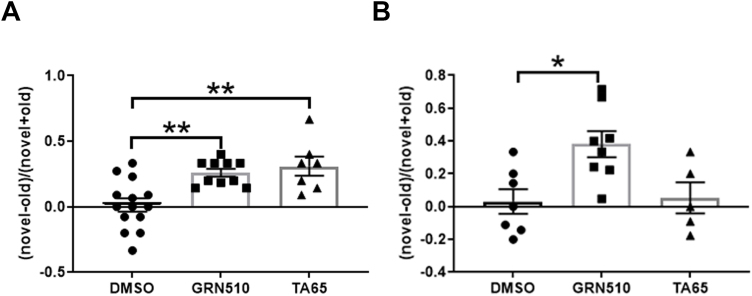


**Figure:**
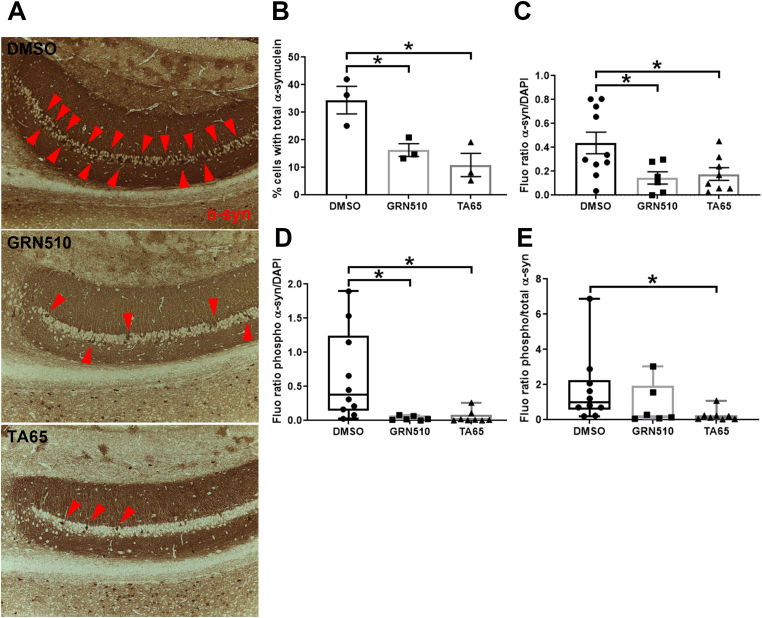


**Figure:**