# Supplementary material for: Increased telomerase improves motor function and alpha-synuclein pathology in a transgenic mouse model of Parkinson’s disease associated with enhanced autophagy
Source: Prog Neurobiol. 2021 Apr;199:101953. doi: 10.1016/j.pneurobio.2020.101953 (PMC7938226; doi:10.1016/j.pneurobio.2020.101953)
Supplement: Supplementary file 1 [file mmc1.docx]

***Supplementary materials***

1. *Supplementary methods:*

*Mice for pilot study*

Female WT mice C57BL/ICFRa (Cameron *et al*., 2011) were used at 24 months. Treatment was for 3 months with 25mg/kg body weight/day TA-65 (Bernardes de Jesus et al., 2011) and 10mg/kg body weight/day GRN510 (Le Saux et al., 2013).

*Telomere in situ hybridization*

Sections were deparaffinised as described previously and then dehydrated again in 70%, 90%, and 100% pre-cooled ethanol at -20°C with 3 minutes for each step. After that, the sections were air-dried for 20 minutes. Subsequently, in situ hybridization was performed with the method described previously (Hallam et al., 2015). Briefly, sections were covered in 10µl hybridization mix and incubated at 80°C for 10 minutes to denature the DNA. The hybridization mix mainly contains 4ng/µl telomere peptide nucleic acid (PNA,) probe (Cy-3 labelled CCCTAA repeats) (Panagene, Korea) and deionized formamide-based hybridization buffer with Blocking Reagent (Roche). Following denaturation, sections were transferred to a dark environment at room temperature and incubated for another 2 hours to facilitate telomere-probe hybridization. Afterwards, the sections were washed in 2X SSC buffer containing 70% formamide, 2X SSC buffer and PBS step by step with 10 minutes for each step. Eventually, the sections were mounted with Vectashield mounting medium with DAPI (Vector Laboratories). Telomere images were captured with a DMi8 fluorescence microscope (Leica, Germany) with Z-stack function activated for obtaining the whole size of telomeres in 3D. All parameters of the microscope were maintained the same when capturing images of all samples. For image analysis, nuclear compact areas of the pyramidal neurons in the CA1 region were manually selected in the DAPI channel and converted into masks, which were then applied to the telomere (red) channel. In the telomere channel, the same brightness and contrast parameter setting was applied to all images to subtract most of the background and to ensure the telomere intensity not overly saturated. The images were converted into 8-bit and the integrated intensity of each telomere particle in the whole CA1 nuclear region, which reflects the quantity of PNA probe binding to each telomere site, was measured by the function of 3D object counter in ImageJ with a threshold particle volume of 0.02-0.5 micron^3^. The average integrated intensity of telomere particles in the whole CA1 region of each animal was calculated blindly and then grouped by treatment after the group information was revealed. Statistics was performed by one-way ANOVA with SigmaPlot 14.0 and the graph generated with GraphPad Prism 8.

*Immuno-histochemistry (IHC) staining*

Paraffin-embedded sections were dewaxed and rehydrated as routinely performed. Antigen retrieval was performed for 10 min of formalin, 15 min in formic acid and then 2 min in a pressure cooker in EDTA at ph 8. Endogenous peroxidase was blocked with 3% hydrogen peroxide for 15 min. An anti-α-synuclein antibody (ab2080, see table 2) was incubated 1:300 at room temperature for 1 h. Signals were detected using the Menarini X-Cell-Plus HRP Detection Kit (Menarini Diagnostics, Winnersh-Wokingham, UK) with 3, 3 diaminobezidine (DAB) as a chromagen and hematoxylin as a counter stain as described previously (McAleese et al., 2019). Stained sections were subsequently dehydrated cleared and mounted using DPX (CellPath, Powys, UK).

*Static rod test*

A metal rod of 2cm diameter with tape around it for better grip and 45 cm length was mounted around 40 cm above bedding material. During the test, mice were placed onto the pole with all paws for 60 s. Time on the rod was recorded, and the procedure repeated five times in total with 1 min resting in between. The average from 5 trials was calculated for each mouse.

*Rearing* test (Fleming et al., 2004, Gellhaar et al., 2015)

Mice were taken to the test room at least 30 minutes prior to the rearing test for habituation. A glass cylinder with a diameter of 15cm was placed into the centre of a wooden platform, and a camera was set up on one side of the platform with a distance to the cylinder to prevent introducing too much distraction to the mice. Video recording was started at the beginning of each test, and then a mouse was carefully transferred into the cylinder. Subsequently, the timing was started with a stopwatch, and the mouse was left undisturbed in the centre of the room during the 5 minutes test time. Between different mice the cylinder was properly cleaned to avoid any odour. Data was collected by counting the rearing numbers from the video captured for each mouse.

*Novel object recognition (NOR) test (Grayson et al., 2015)*

Mice were allowed to habituate in the testing cage for 15 minutes on two consecutive days. On the testing day mice were taken to the test room at least 30 minutes prior to the test for habituating the testing environment. The test consisted of two parts for each mouse with a time gap of 15 minutes in between. In the first part mice were trained for 2 identical “old” objects at a 20 cm distance from each other. Each mouse was given 3 minutes in the cage to explore the objects and the test video recorded. In the second part, one of the cleaned “old” objects and a “novel” odourless object with a different shape were placed in a clean cage and video recorded. For analysis, the number of contacts (less than 5mm distance between mouse and object without counting any sitting on the object) with the “old” and “novel” objects of each mouse was counted from the video captured for the second section. Then the result was calculated as a ratio of (novel object contacts – old object contacts)/(novel object contacts + old object contacts).

*Determination of protein content in brain lysates*

From frozen brain tissue between 20 and 60mg were lysed in a suitable volume of RIPA buffer (around 400μl) and protein content (μg/μl) determined using a Bradford assay (Bio-Rad) according to manufacturer’s instructions. From those values the protein amount per mg brain tissue was calculated.

*Primary antibodies for proteins*

| Antibody name | Species | Dilution for IF | Manufacturer, number |
| --- | --- | --- | --- |
| Anti-MnSOD | rabbit | 1:100 | Millipore 06-984 |
| Anti-MTCO1 | mouse | 1:1000 | Abcam, ab14705 |
| Anti-Tyrosine hydroxylase | Rabbit | 1:1000 | Abcam, ab112 |
| Anti-β-III tubulin | Rabbit | 1:500 | Abcam, ab18207 |

***References:***

Cameron, K.M., Golightly, A., Miwa, S., Speakman, J., Boys, R., von Zglinicki, T. 2011. [Gross energy metabolism in mice under late onset, short term caloric restriction.](https://www.ncbi.nlm.nih.gov/pubmed/21507329) Mech Ageing Dev. 132(4):202-209. http://doi: 10.1016/j.mad.2011.04.004.

[Fleming SM](https://www.ncbi.nlm.nih.gov/pubmed/?term=Fleming%20SM%5BAuthor%5D&cauthor=true&cauthor_uid=15496679), [Salcedo J](https://www.ncbi.nlm.nih.gov/pubmed/?term=Salcedo%20J%5BAuthor%5D&cauthor=true&cauthor_uid=15496679), [Fernagut PO](https://www.ncbi.nlm.nih.gov/pubmed/?term=Fernagut%20PO%5BAuthor%5D&cauthor=true&cauthor_uid=15496679), [Rockenstein E](https://www.ncbi.nlm.nih.gov/pubmed/?term=Rockenstein%20E%5BAuthor%5D&cauthor=true&cauthor_uid=15496679), [Masliah E](https://www.ncbi.nlm.nih.gov/pubmed/?term=Masliah%20E%5BAuthor%5D&cauthor=true&cauthor_uid=15496679), [Levine MS](https://www.ncbi.nlm.nih.gov/pubmed/?term=Levine%20MS%5BAuthor%5D&cauthor=true&cauthor_uid=15496679) Chesselet, M.F*.* 2004. Early and progressive sensorimotor anomalies in mice overexpressing wild-type human alpha-synuclein. [J Neurosci.](https://www.ncbi.nlm.nih.gov/pubmed/?term=Fleming+2004+Thy) 24(42):9434-40. http://doi: 10.1523/JNEUROSCI.3080-04.2004.

Gellhaar S, Marcellino D, Abrams MB, Galter D. 2015. [Chronic L-DOPA induces hyperactivity, ormalization of gait and dyskinetic behavior in MitoPark mice.](https://www.ncbi.nlm.nih.gov/pubmed/25752644) Genes Brain Behav. 14(3):260-70.

[Grayson, B](https://www.ncbi.nlm.nih.gov/pubmed/?term=Grayson%20B%5BAuthor%5D&cauthor=true&cauthor_uid=25447293)., [Leger, M](https://www.ncbi.nlm.nih.gov/pubmed/?term=Leger%20M%5BAuthor%5D&cauthor=true&cauthor_uid=25447293)., [Piercy, C](https://www.ncbi.nlm.nih.gov/pubmed/?term=Piercy%20C%5BAuthor%5D&cauthor=true&cauthor_uid=25447293)., [Adamson, L](https://www.ncbi.nlm.nih.gov/pubmed/?term=Adamson%20L%5BAuthor%5D&cauthor=true&cauthor_uid=25447293)., [Harte, M](https://www.ncbi.nlm.nih.gov/pubmed/?term=Harte%20M%5BAuthor%5D&cauthor=true&cauthor_uid=25447293)., [Neill, J.C](https://www.ncbi.nlm.nih.gov/pubmed/?term=Neill%20JC%5BAuthor%5D&cauthor=true&cauthor_uid=25447293). 2015. Assessment of disease-related cognitive impairments using the novel object recognition (NOR) task in rodents. [Behav Brain Res.](https://www.ncbi.nlm.nih.gov/pubmed/25447293) 5 285:176-93. http://doi: 10.1016/j.bbr.2014.10.025.

Hallam, D., Wan, T., Saretzki, G. Dietary restriction mitigates age-related accumulation of DNA damage, but not all changes in mouse corneal epithelium. Exp Gerontol. 2015 67:72-79. http://doi: 10.1016/j.exger.2015.04.014.

McAleese, K.E., Graham, S., Dey, M., Walker, L., Erskine, D., Johnson, M., Johnston, E., Thomas, A.J., McKeith, I.G., DeCarli, C., Attems, J.2019. [Extravascular fibrinogen in the white matter of Alzheimer's disease and normal aged brains: implications for fibrinogen as a biomarker for Alzheimer's disease.](https://www.ncbi.nlm.nih.gov/pubmed/30485582) Brain Pathol.29(3):414-24. http://doi: 10.1111/bpa.12685.

1. ***Supplementary figures***

**Fig. S1:  *Tert* expression in brains and balance of two year old WT mice and cultured mouse neurons after treatment with telomerase activators**

**A**: *Tert* expression in the brains of 27 months old mice treated with each telomerase activator or DMSO for 3 months. Bars represent mean±SE and sample size is 5, 3, and 5 for DMSO, GRN510 and TA65 groups, respectively. The P value for each of the treatment group compared to the DMSO control group is p<0.001. Results are presented as 2^-ΔΔCT^ and statistical analysis was performed by one way ANOVA a Holm-Sidak as post-hoc test. **B:** Balance on a static rod increases in 27 months old wild-type mice after treatment with both telomerase activators. Bars represent mean±SE old DMSO: n=8, TA65: n=7, GRN510: n=5, young wild-type mice (3 months) n=7. Statistical analysis was performed using a ANOVA on ranks with Dunn’s post-hoc test. * p<0.05, ** p<0.01, ***p<0.001,

**C:** qPCR data of *Tert* expression in neurons treated with GRN510 (500 nM) and in the matched DMSO control (n=5). The data show median and dots of data from minimum to maximum analysed by t-test on ranks. **D:** *Tert* expression in neurons under TA65 treatment (10 μM) compared to matched DMSO controls from 3 biological repeats. Data show mean±SEM of (n=3) analysed by two-tailed t-test . ** p<0.01,

**Fig. S2: Telomere length of pyramidal neurons in hippocampal CA1**

Telomere length expressed as integrated intensity of the telomere signal after hybridisation with a telomere probe in DMSO, GRN510 and TA-65 treated mice. n=9, 6, 8, respectively. Kruskal-Wallis One Way Analysis of Variance on Ranks P=0.334 (not significant)

**Fig. S3: No weight changes due to treatments**

Shown are the results of mouse weights at 18 months. **A:** in females: N=14 for control, n=10 for GRN510, n=7 for TA65 **B:** in males, n= 7 for control, 8 for GRN, n=5 for TA65,

**Fig. S4: Rearing test of telomerase activator treated line D mice**

Data in all graphs present means ± SEM of rearing numbers with both forelimbs.

**A:** females DMSO n=14; GRN510 n=10 TA65 n=7. **B:** males DMSO n=7, GRN510 n=8, TA65 n=5, * p<0.05,

**Fig. S5: Novel object recognition test of line D mice from after telomerase activator treatment**

**A:** Females. DMSO n=14; GRN510 n=10 TA65 n=7. **B:** Males. DMSO n=7, GRN510 n=8, TA65 n=5. Data are presented as mean ± SEM. Statistics was performed using One way ANOVA with Holm-Sidak post-hoc test. * p<0.05, ** p<0.01,

**Fig. S6: α-syn positive cells analysed only in the pyramidal neuronal layer of hippocampal CA1**

**A:** Representative images of **immunohistochemistry (**IHC) staining of t**otal human α-synuclein (brown) in the hippocampus CA1 region of line D mice under different treatments. Red arrows point to the layer of pyramidal neurons.**

**B:** Quantification of percentage of stained α-synuclein positive cells among all pyramidal neurons in CA1, Sample size: n=3 for each group. **C, D, E: Total and phosphorylated α-synuclein levels in the neuronal body area of the CA1 pyramidal neuron layer from IF staining** (see Fig. 5 A) **C:** relative levels of total α-synuclein in the selected area normalised to total nucleus numbers using DAPI staining. Statistics was performed using a One way ANOVA test, Bars present mean± SEM, **D:** relative levels of phosphorylated α-synuclein in the pyramidal layer normalised to total nucleus numbers using DAPI staining (see Fig. 5 A). Statistics was performed using ANOVA on ranks. **E:** shows the intensity ratio of phosphorylated/total α-synuclein in the hippocampal pyramidal layer, statistics was performed using ANOVA on ranks. Data in D and E are not normally distributed and are presented in Box & Whiskers plots as median with minimum to maximum. Sample size: DMSO n=10, GRN510 n=6, and TA65 n=8. * p<0.05,

**Fig. S7 No changes in protein content of various proteins in treated brains**

**A:** Protein amount per mg brain tissue from DMSO controls (n=5), GRN 510 (n=5) and TA-65 (n=4) treated mice. **B:** MnSOD/DAPI fluorescence ratio in the neocortex. **C:** MTCO1/DAPI fluorescence ratio in the neocortex. **D:** Tyrosine hydroxylase/DAPI fluorescence ratio in the striatum. n=8, 6, and 8 for DMSO, GRN510 and TA65 groups, respectively. **E:** β-III tubulin/DAPI fluorescence ratio in CA1. n=9, 6, and 8 for DMSO, GRN510 and TA65 groups respectively. No significant differences in any of the graphs (analysed with ANOVA)
